# Crystal structure of ethyl 2-chloro-5,8-di­meth­oxy­quinoline-3-carboxyl­ate

**DOI:** 10.1107/S1600536814017309

**Published:** 2014-08-01

**Authors:** Hasna Hayour, Abdelmalek Bouraiou, Sofiane Bouacida, Saida Benzerka, Ali Belfaitah

**Affiliations:** a Laboratoire des Produits Naturels d’Origine Végétale et de Synthèse Organique, PHYSYNOR, Université Constantine 1, 25000 Constantine, Algeria; b Unité de Recherche de Chimie de l’Environnement et Moléculaire Structurale (CHEMS), Université Constantine 1, 25000 , Algeria; c Département Sciences de la Matière, Faculté des Sciences Exactes et Sciences de la Nature et de la Vie, Université Oum El Bouaghi, Algeria

**Keywords:** crystal structure, quinoline derivatives, ester, hydrogen bonding, π–π stacking

## Abstract

In the title compound, C_14_H_14_ClNO_4_, the dihedral angle between the quinoline ring system (r.m.s. deviation = 0.0142 Å) and ester planes is 18.99 (3)°. The C—O—C—C_m_ (m = meth­yl) torsion angle is −172.08 (10)°, indicating a *trans* conformation. In the crystal, the mol­ecules are linked by C—H⋯O and C—H⋯N inter­actions, generating layers lying parallel to (101). Aromatic π-π stacking [centroid–centroid distances = 3.557 (2) and 3.703 (2)Å] links the layers into a three-dimensional network.

## Related literature   

For the synthesis and applications of quinoline derivatives, see: Wang *et al.* (2011 ▶); Benzerka *et al.* (2012 ▶); Valdez *et al.* (2009 ▶). For our previous work, see: Bouraiou *et al.* (2012 ▶); Hayour *et al.* (2014 ▶); Benzerka *et al.* (2012 ▶).
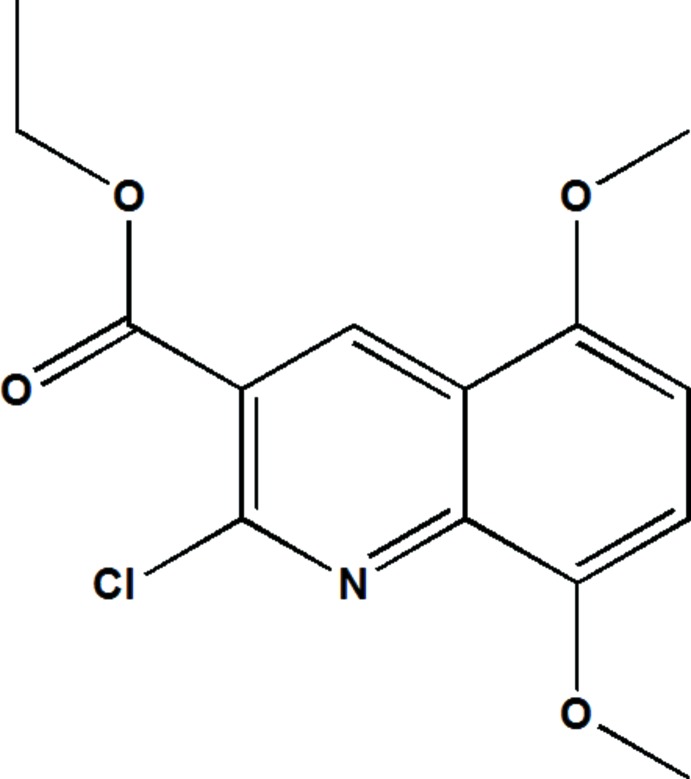


## Experimental   

### Crystal data  

C_14_H_14_ClNO_4_
*M* *_r_* = 295.71
Triclinic, 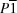

*a* = 7.512 (4) Å
*b* = 9.759 (5) Å
*c* = 9.811 (5) Å
α = 76.071 (10)°
β = 72.021 (10)°
γ = 86.037 (10)°
*V* = 664.0 (6) Å^3^
*Z* = 2
Mo *K*α radiation
μ = 0.30 mm^−1^
*T* = 150 K
0.25 × 0.14 × 0.12 mm

### Data collection  

Bruker APEXII diffractometer
Absorption correction: multi-scan (*SADABS*; Sheldrick, 2002 ▶) *T*_min_ = 0.690, *T*_max_ = 0.747
10769 measured reflections
5204 independent reflections
4090 reflections with *I* > 2σ(*I*)
*R*_int_ = 0.024

### Refinement  

*R*[*F* ^2^ > 2σ(*F* ^2^)] = 0.036
*wR*(*F* ^2^) = 0.103
*S* = 1.04
5204 reflections
184 parameters
H-atom parameters constrained
Δρ_max_ = 0.5 e Å^−3^
Δρ_min_ = −0.24 e Å^−3^

### 

Data collection: *APEX2* (Bruker, 2006 ▶); cell refinement: *SAINT* (Bruker, 2006 ▶); data reduction: *SAINT*; program(s) used to solve structure: *SIR2002* (Burla *et al.*, 2005 ▶); program(s) used to refine structure: *SHELXL97* (Sheldrick, 2008 ▶); molecular graphics: *ORTEP-3 for Windows* (Farrugia, 2012 ▶) and *DIAMOND* (Brandenburg & Berndt, 2001 ▶); software used to prepare material for publication: *WinGX* (Farrugia, 2012 ▶).

## Supplementary Material

Crystal structure: contains datablock(s) I. DOI: 10.1107/S1600536814017309/hg5402sup1.cif

Structure factors: contains datablock(s) I. DOI: 10.1107/S1600536814017309/hg5402Isup2.hkl

Supporting information file. DOI: 10.1107/S1600536814017309/hg5402Isup3.cml

**Fig. 1.**
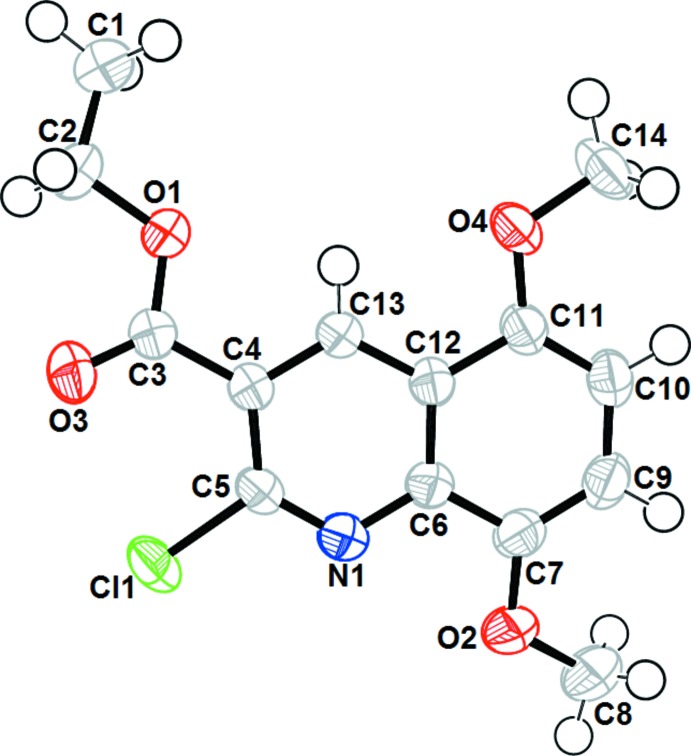
(Farrugia, 2012) the structure of the title compound with the atomic labelling scheme. Displacement are drawn at the 50% probability level.

**Fig. 2.**
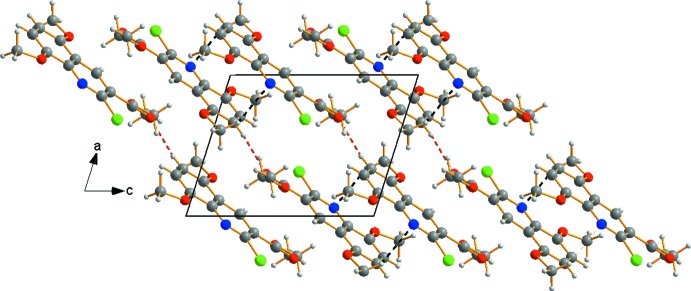
(Brandenburg & Berndt, 2001) A diagram of the layered crystal packing of (I) viewed down the b axis and showing hydrogen bond [C—H···O in red and C—H···N in black] as dashed line.

. DOI: 10.1107/S1600536814017309/hg5402fig1.tif
(Farrugia, 2012) the structure of the title compound with the atomic labelling scheme. Displacement are drawn at the 50% probability level.

b . DOI: 10.1107/S1600536814017309/hg5402fig2.tif
(Brandenburg & Berndt, 2001) A diagram of the layered crystal packing of (I) viewed down the *b* axis and showing hydrogen bond [C—H⋯O in red and C—H⋯N in black] as dashed line.

CCDC reference: 1016211

Additional supporting information:  crystallographic information; 3D view; checkCIF report

## Figures and Tables

**Table 1:** Hydrogen-bond geometry (Å, °)

*D*—H⋯*A*	*D*—H	H⋯*A*	*D*⋯*A*	*D*—H⋯*A*
C10—H10⋯O3^i^	0.93	2.56	3.482 (2)	173
C14—H14*C*⋯N1^ii^	0.96	2.61	3.476 (2)	150

Symmetry codes: (i) 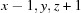; (ii) 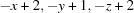.

## supplementary crystallographic information

### S1. Comment

Quinolines have attracted considerable interest for many years due to their presence in the skeleton of a large number of bioactive compounds and natural products (Wang, *et al.* 2011), such as antibacterial (Benzerka, *et al.*2012). in going with our investigation, recently, we have reported the synthesis and structure determination of some new quinoline compounds (Hayour, *et al.*, 2014; Bouraiou, *et al.* 2012). In this paper, we describe the synthesis and the structure determination of ethyl 2-chloro-5,8-dimethoxyquinoline-3-carboxylate (I) which obtained in one step, by addition of NaCN in presence of manganese dioxide in absolute ethanol to 2-chloro-5,8-dimethoxyquinoline-3-carbaldehyde. The molecular geometry and the atom-numbering scheme of (I) are shown in Fig. 1. In the asymmetric unit of title compound the quinoline ring is four time substituted by two methoxy, one chlore and one ethyl carboxylate. The two rings of quinolyl moiety are fused in an axial fashion and form dihedral angle of 1.75 (3) Å. The crystal packing can be described as double layers parallel to (101) plane, along the *b* axis (Fig. 2). It is stabilized by intermolecular hydrogen bond (N—H···O and C—H···O) and strong π–π stacking, resulting in the formation of infinite a three-dimensional network linking these layers together and reinforces cohesion of the structure (Fig. 2). Hydrogen-bonding parameters are listed in Table 1.

### S2. Experimental

To a cold solution of NaCN (3 mmol) in absolute ethanol (15 mL), a mixture of 2-chloro-5,8-dimethoxy quinolin-3-carbaldehyde (1 mmol) and manganese dioxide (6.7 mmol) was added at 0°C, then the reaction mixture was stirred at 25°C during 3 h. After complexion, the title compound was obtained by simple filtration through a small column packed with 4 cm of celite and 3 cm of silica gel using CH_2_Cl_2_ as eluant (Valdez, *et al.* 2009).

### S3. Refinement

All non-H atoms were refined with anisotropic atomic displacement parameters. All H atoms were localized on Fourier maps but introduced in calculated positions and treated as riding on their parent C atom. (with C—H = 0.93 (aromatic), 0.96 (methyl) and 0.97 Å (methylene) and *U*_iso_(H) = 1.5 or 1.2 (carrier atom).

### Crystal data

**Table:**

C_14_H_14_ClNO_4_	*Z* = 2
*M**_r_* = 295.71	*F*(000) = 308
Triclinic, *P*1	*D*_x_ = 1.479 Mg m^−^^3^
Hall symbol: -P 1	Mo *K*α radiation, λ = 0.71073 Å
*a* = 7.512 (4) Å	Cell parameters from 4109 reflections
*b* = 9.759 (5) Å	θ = 2.7–34.1°
*c* = 9.811 (5) Å	µ = 0.30 mm^−^^1^
α = 76.071 (10)°	*T* = 150 K
β = 72.021 (10)°	Prism, colorless
γ = 86.037 (10)°	0.25 × 0.14 × 0.12 mm
*V* = 664.0 (6) Å^3^	

### Data collection

**Table:**

Bruker APEXII diffractometer	4090 reflections with *I* > 2σ(*I*)
Graphite monochromator	*R*_int_ = 0.024
CCD rotation images, thin slices scans	θ_max_ = 34.7°, θ_min_ = 2.7°
Absorption correction: multi-scan (*SADABS*; Sheldrick, 2002)	*h* = −11→11
*T*_min_ = 0.690, *T*_max_ = 0.747	*k* = −15→15
10769 measured reflections	*l* = −15→15
5204 independent reflections	

### Refinement

**Table:**

Refinement on *F*^2^	Primary atom site location: structure-invariant direct methods
Least-squares matrix: full	Secondary atom site location: difference Fourier map
*R*[*F*^2^ > 2σ(*F*^2^)] = 0.036	Hydrogen site location: inferred from neighbouring sites
*wR*(*F*^2^) = 0.103	H-atom parameters constrained
*S* = 1.04	*w* = 1/[σ^2^(*F*_o_^2^) + (0.0528*P*)^2^ + 0.079*P*] where *P* = (*F*_o_^2^ + 2*F*_c_^2^)/3
5204 reflections	(Δ/σ)_max_ = 0.001
184 parameters	Δρ_max_ = 0.5 e Å^−^^3^
0 restraints	Δρ_min_ = −0.24 e Å^−^^3^

### Special details

**Table:**

Geometry. All e.s.d.'s (except the e.s.d. in the dihedral angle between two l.s. planes) are estimated using the full covariance matrix. The cell e.s.d.'s are taken into account individually in the estimation of e.s.d.'s in distances, angles and torsion angles; correlations between e.s.d.'s in cell parameters are only used when they are defined by crystal symmetry. An approximate (isotropic) treatment of cell e.s.d.'s is used for estimating e.s.d.'s involving l.s. planes.
Refinement. Refinement of *F*^2^ against ALL reflections. The weighted *R*-factor *wR* and goodness of fit *S* are based on *F*^2^, conventional *R*-factors *R* are based on *F*, with *F* set to zero for negative *F*^2^. The threshold expression of *F*^2^ > σ(*F*^2^) is used only for calculating *R*-factors(gt) *etc*. and is not relevant to the choice of reflections for refinement. *R*-factors based on *F*^2^ are statistically about twice as large as those based on *F*, and *R*- factors based on ALL data will be even larger.

### Fractional atomic coordinates and isotropic or equivalent isotropic displacement parameters (Å^2^)

**Table:**

	*x*	*y*	*z*	*U*_iso_*/*U*_eq_	
C1	1.27548 (18)	1.02335 (11)	0.38370 (13)	0.0317 (2)	
H1B	1.3474	1.0184	0.4504	0.048*	
H1A	1.1478	1.0451	0.4304	0.048*	
H1C	1.3262	1.0958	0.2965	0.048*	
C2	1.28321 (17)	0.88354 (11)	0.34275 (11)	0.0266 (2)	
H2A	1.2118	0.8873	0.2747	0.032*	
H2B	1.4116	0.8599	0.2961	0.032*	
C3	1.21881 (13)	0.64349 (10)	0.46758 (10)	0.01689 (16)	
C4	1.11813 (13)	0.54830 (9)	0.61114 (10)	0.01547 (15)	
C5	1.14674 (13)	0.39997 (10)	0.64961 (10)	0.01641 (16)	
C6	0.92888 (13)	0.37156 (9)	0.87752 (10)	0.01595 (16)	
C7	0.83649 (14)	0.28087 (10)	1.01633 (10)	0.01932 (17)	
C8	0.82466 (19)	0.05352 (12)	1.17645 (13)	0.0316 (2)	
H8A	0.8799	−0.0381	1.176	0.047*	
H8B	0.6908	0.0448	1.2073	0.047*	
H8C	0.8618	0.0943	1.2434	0.047*	
C9	0.70785 (14)	0.33822 (11)	1.12113 (10)	0.02025 (18)	
H9	0.6474	0.2795	1.2116	0.024*	
C10	0.66446 (13)	0.48421 (11)	1.09552 (10)	0.01941 (17)	
H10	0.5761	0.5196	1.1683	0.023*	
C11	0.75286 (13)	0.57316 (10)	0.96332 (10)	0.01729 (16)	
C12	0.88663 (12)	0.51757 (9)	0.85187 (10)	0.01559 (16)	
C13	0.98519 (13)	0.60393 (9)	0.71557 (10)	0.01569 (16)	
H13	0.9603	0.7001	0.6955	0.019*	
C14	0.61166 (16)	0.78024 (12)	1.03759 (11)	0.0250 (2)	
H14A	0.4863	0.7439	1.0691	0.038*	
H14B	0.6108	0.8806	0.9999	0.038*	
H14C	0.661	0.7598	1.1196	0.038*	
N1	1.05905 (11)	0.31542 (8)	0.77389 (9)	0.01767 (15)	
O1	1.20348 (11)	0.77897 (8)	0.47853 (8)	0.02506 (16)	
O2	0.88630 (12)	0.14209 (8)	1.03191 (8)	0.02716 (17)	
O3	1.30129 (11)	0.60788 (8)	0.35513 (8)	0.02479 (16)	
O4	0.72630 (11)	0.71537 (8)	0.92457 (8)	0.02404 (16)	
Cl1	1.31414 (4)	0.31664 (3)	0.52818 (3)	0.02578 (7)	

### Atomic displacement parameters (Å^2^)

**Table:**

	*U*^11^	*U*^22^	*U*^33^	*U*^12^	*U*^13^	*U*^23^
C1	0.0359 (6)	0.0187 (5)	0.0298 (5)	−0.0004 (4)	0.0026 (5)	−0.0019 (4)
C2	0.0336 (6)	0.0190 (4)	0.0186 (4)	−0.0029 (4)	0.0014 (4)	0.0003 (3)
C3	0.0171 (4)	0.0168 (4)	0.0160 (4)	−0.0001 (3)	−0.0032 (3)	−0.0043 (3)
C4	0.0161 (4)	0.0154 (4)	0.0144 (4)	0.0003 (3)	−0.0030 (3)	−0.0044 (3)
C5	0.0173 (4)	0.0158 (4)	0.0164 (4)	0.0020 (3)	−0.0038 (3)	−0.0064 (3)
C6	0.0172 (4)	0.0150 (4)	0.0156 (4)	−0.0006 (3)	−0.0043 (3)	−0.0040 (3)
C7	0.0221 (4)	0.0163 (4)	0.0184 (4)	−0.0029 (3)	−0.0048 (3)	−0.0028 (3)
C8	0.0421 (7)	0.0189 (5)	0.0245 (5)	−0.0025 (4)	−0.0018 (5)	0.0024 (4)
C9	0.0207 (4)	0.0213 (4)	0.0160 (4)	−0.0050 (3)	−0.0021 (3)	−0.0024 (3)
C10	0.0176 (4)	0.0231 (4)	0.0160 (4)	−0.0005 (3)	−0.0017 (3)	−0.0060 (3)
C11	0.0174 (4)	0.0179 (4)	0.0157 (4)	0.0019 (3)	−0.0033 (3)	−0.0051 (3)
C12	0.0153 (4)	0.0163 (4)	0.0144 (4)	−0.0002 (3)	−0.0030 (3)	−0.0041 (3)
C13	0.0170 (4)	0.0140 (4)	0.0149 (4)	0.0006 (3)	−0.0030 (3)	−0.0036 (3)
C14	0.0278 (5)	0.0267 (5)	0.0207 (4)	0.0105 (4)	−0.0046 (4)	−0.0120 (4)
N1	0.0199 (4)	0.0154 (3)	0.0174 (3)	0.0006 (3)	−0.0046 (3)	−0.0047 (3)
O1	0.0331 (4)	0.0153 (3)	0.0183 (3)	−0.0012 (3)	0.0037 (3)	−0.0026 (2)
O2	0.0384 (4)	0.0146 (3)	0.0212 (3)	−0.0004 (3)	−0.0009 (3)	−0.0010 (3)
O3	0.0300 (4)	0.0243 (4)	0.0163 (3)	−0.0032 (3)	0.0012 (3)	−0.0075 (3)
O4	0.0294 (4)	0.0186 (3)	0.0183 (3)	0.0073 (3)	0.0005 (3)	−0.0054 (3)
Cl1	0.02904 (13)	0.02190 (12)	0.02243 (12)	0.00813 (9)	−0.00059 (9)	−0.00934 (9)

### Geometric parameters (Å, º)

**Table:**

C1—C2	1.5050 (17)	C7—C9	1.3760 (14)
C1—H1B	0.96	C8—O2	1.4257 (14)
C1—H1A	0.96	C8—H8A	0.96
C1—H1C	0.96	C8—H8B	0.96
C2—O1	1.4534 (13)	C8—H8C	0.96
C2—H2A	0.97	C9—C10	1.4189 (15)
C2—H2B	0.97	C9—H9	0.93
C3—O3	1.2062 (12)	C10—C11	1.3722 (14)
C3—O1	1.3463 (13)	C10—H10	0.93
C3—C4	1.4928 (13)	C11—O4	1.3656 (13)
C4—C13	1.3796 (13)	C11—C12	1.4254 (13)
C4—C5	1.4241 (14)	C12—C13	1.4068 (13)
C5—N1	1.3025 (13)	C13—H13	0.93
C5—Cl1	1.7500 (10)	C14—O4	1.4301 (12)
C6—N1	1.3677 (12)	C14—H14A	0.96
C6—C12	1.4173 (14)	C14—H14B	0.96
C6—C7	1.4286 (14)	C14—H14C	0.96
C7—O2	1.3650 (14)		
			
C2—C1—H1B	109.5	H8A—C8—H8B	109.5
C2—C1—H1A	109.5	O2—C8—H8C	109.5
H1B—C1—H1A	109.5	H8A—C8—H8C	109.5
C2—C1—H1C	109.5	H8B—C8—H8C	109.5
H1B—C1—H1C	109.5	C7—C9—C10	122.07 (9)
H1A—C1—H1C	109.5	C7—C9—H9	119
O1—C2—C1	106.85 (9)	C10—C9—H9	119
O1—C2—H2A	110.4	C11—C10—C9	120.01 (9)
C1—C2—H2A	110.4	C11—C10—H10	120
O1—C2—H2B	110.4	C9—C10—H10	120
C1—C2—H2B	110.4	O4—C11—C10	126.18 (8)
H2A—C2—H2B	108.6	O4—C11—C12	114.27 (8)
O3—C3—O1	123.25 (9)	C10—C11—C12	119.55 (9)
O3—C3—C4	126.28 (9)	C13—C12—C6	117.50 (8)
O1—C3—C4	110.46 (8)	C13—C12—C11	122.18 (9)
C13—C4—C5	116.02 (8)	C6—C12—C11	120.29 (8)
C13—C4—C3	119.42 (9)	C4—C13—C12	121.12 (9)
C5—C4—C3	124.56 (8)	C4—C13—H13	119.4
N1—C5—C4	125.27 (8)	C12—C13—H13	119.4
N1—C5—Cl1	114.12 (7)	O4—C14—H14A	109.5
C4—C5—Cl1	120.60 (7)	O4—C14—H14B	109.5
N1—C6—C12	121.71 (8)	H14A—C14—H14B	109.5
N1—C6—C7	118.96 (9)	O4—C14—H14C	109.5
C12—C6—C7	119.31 (8)	H14A—C14—H14C	109.5
O2—C7—C9	125.81 (9)	H14B—C14—H14C	109.5
O2—C7—C6	115.43 (9)	C5—N1—C6	118.38 (8)
C9—C7—C6	118.76 (9)	C3—O1—C2	115.86 (8)
O2—C8—H8A	109.5	C7—O2—C8	116.99 (8)
O2—C8—H8B	109.5	C11—O4—C14	116.88 (8)
			
O3—C3—C4—C13	−160.43 (10)	C7—C6—C12—C11	−0.20 (13)
O1—C3—C4—C13	18.58 (12)	O4—C11—C12—C13	1.56 (13)
O3—C3—C4—C5	18.55 (16)	C10—C11—C12—C13	−178.38 (9)
O1—C3—C4—C5	−162.43 (9)	O4—C11—C12—C6	179.62 (8)
C13—C4—C5—N1	−0.45 (14)	C10—C11—C12—C6	−0.32 (14)
C3—C4—C5—N1	−179.46 (9)	C5—C4—C13—C12	0.60 (13)
C13—C4—C5—Cl1	−178.93 (7)	C3—C4—C13—C12	179.67 (8)
C3—C4—C5—Cl1	2.06 (13)	C6—C12—C13—C4	−0.11 (13)
N1—C6—C7—O2	−1.04 (13)	C11—C12—C13—C4	178.00 (9)
C12—C6—C7—O2	−179.64 (8)	C4—C5—N1—C6	−0.23 (14)
N1—C6—C7—C9	178.96 (9)	Cl1—C5—N1—C6	178.33 (7)
C12—C6—C7—C9	0.36 (14)	C12—C6—N1—C5	0.77 (13)
O2—C7—C9—C10	179.98 (9)	C7—C6—N1—C5	−177.79 (9)
C6—C7—C9—C10	−0.02 (15)	O3—C3—O1—C2	3.72 (15)
C7—C9—C10—C11	−0.50 (15)	C4—C3—O1—C2	−175.33 (8)
C9—C10—C11—O4	−179.27 (9)	C1—C2—O1—C3	−172.08 (10)
C9—C10—C11—C12	0.66 (14)	C9—C7—O2—C8	−11.40 (15)
N1—C6—C12—C13	−0.60 (13)	C6—C7—O2—C8	168.60 (9)
C7—C6—C12—C13	177.95 (9)	C10—C11—O4—C14	7.48 (15)
N1—C6—C12—C11	−178.76 (9)	C12—C11—O4—C14	−172.46 (9)

### Hydrogen-bond geometry (Å, º)

**Table:**

*D*—H···*A*	*D*—H	H···*A*	*D*···*A*	*D*—H···*A*
C10—H10···O3^i^	0.93	2.56	3.482 (2)	173
C14—H14*C*···N1^ii^	0.96	2.61	3.476 (2)	150
C13—H13···O1	0.93	2.34	2.6713 (19)	101
C13—H13···O4	0.93	2.42	2.7366 (19)	100

Symmetry codes: (i) *x*−1, *y*, *z*+1; (ii) −*x*+2, −*y*+1, −*z*+2.
