# Supplementary figures and images for: Crystal structure of ethyl 2-chloro-5,8-di­meth­oxy­quinoline-3-carboxyl­ate
Source: Acta Crystallogr Sect E Struct Rep Online. 2014 Aug 1;70(Pt 9):o964–5. doi: 10.1107/S1600536814017309 (PMC4186085; doi:10.1107/S1600536814017309)

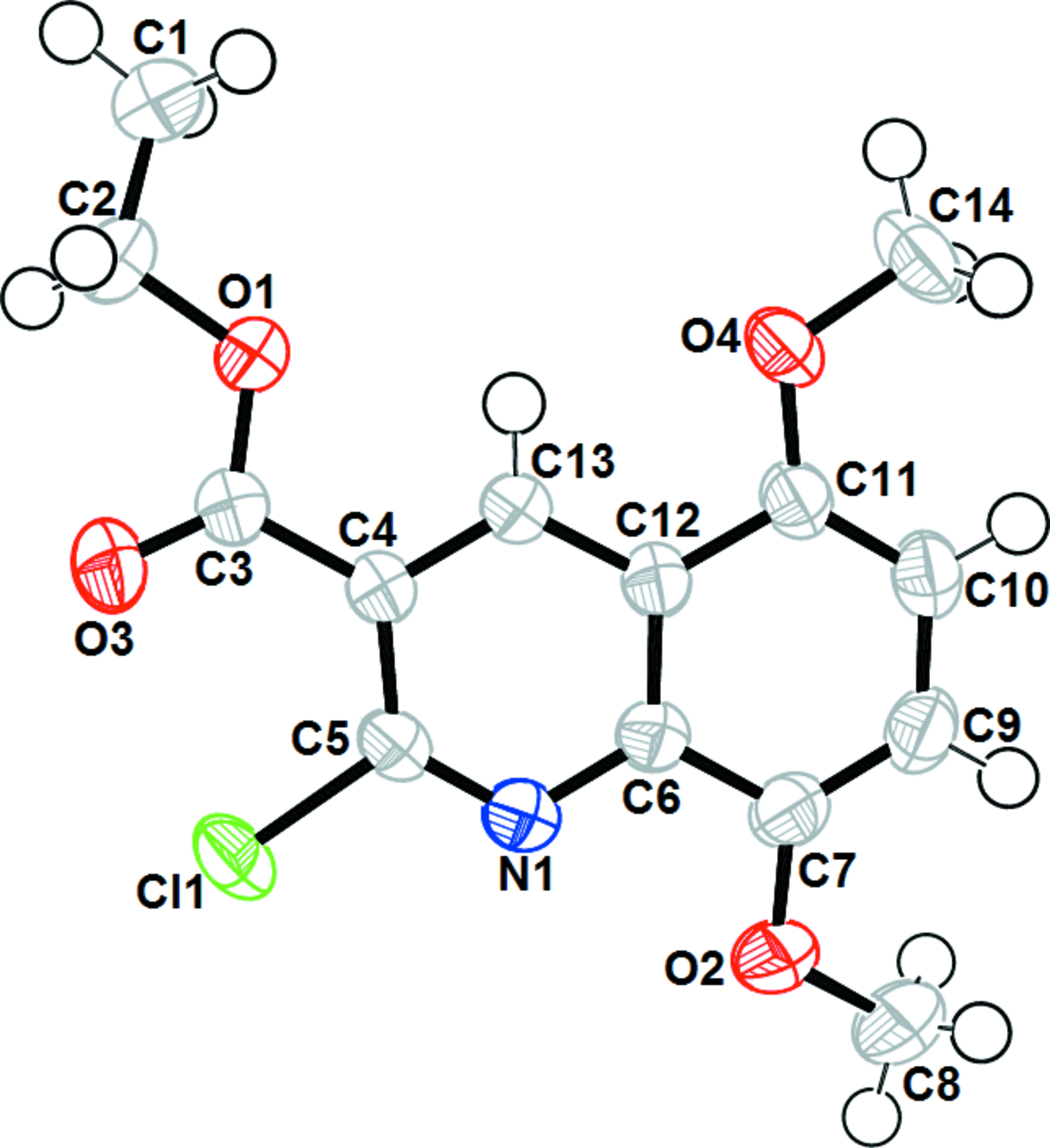

Supplement: Supplementary file 4 [file e-70-0o964-fig1.tif]

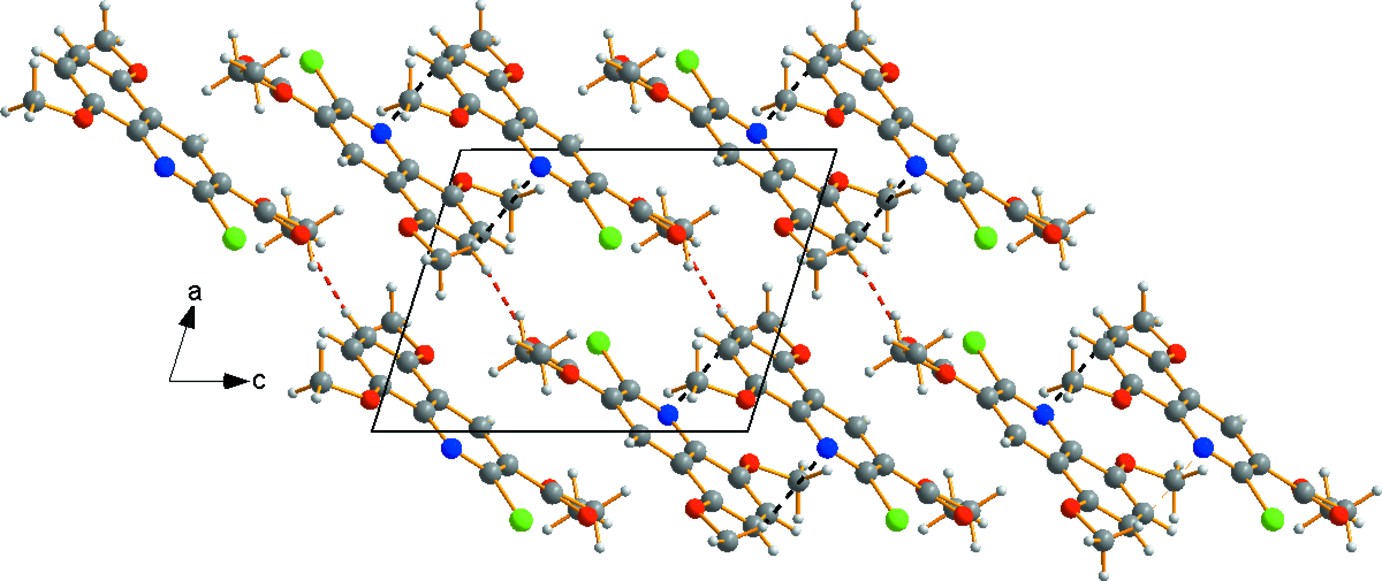

Supplement: Supplementary file 5 [file e-70-0o964-fig2.tif]
